# Supplementary material for: Prognostic value of postoperative serum carcinoembryonic antigen levels in colorectal cancer patients who smoke
Source: PLoS One. 2020 Jun 5;15(6):e0233687. doi: 10.1371/journal.pone.0233687 (PMC7274431; doi:10.1371/journal.pone.0233687)
Supplement: Attachment — Submitted filename: My commend.docx [file pone.0233687.s002.docx]

In this study, authors aimed to evaluate the impact of smoking on the prognostic value of serum CEA levels. In this single site, they retrospectively collected clinical data, treatment outcomes, smoking status and CEA level before and after operation. They found that although patients whose CEA levels were normal preoperative and postoperative showed the best 3-year DFS, elevated postoperative CEA levels were associated with lower DFS rates in never and former smokers, but were not associated with lower DFS rates in current smokers.

The strength of this study is that they found that persistent smoking altered the prognostic value of postoperative serum CEA levels in colorectal cancer patients. This is the first study to determine the prognostic value of CEA status in smoking and nonsmoking patients with colorectal cancer. Before accepted for publication, they are still some issues.

Major issue

1. Different stage will lead to different outcome. In colon cancer, molecular markers such as K-Ras,N-Ras and B-Raf are also an important factors. This study included stage I, II and III colon-rectal cancer. Authors should list the distribution of stages within different groups. If possible, analysis of molecular subtypes are also important.
2. In line 223, authors mentioned about groups C had the worse outcome. But in group C, there were more patients with right side tumors compared with Group B and A, and more patients were LN positive compared with Group A. In summery, group C was already with worse prognostic factors, nothing to do with CEA level before and after operation. Interesting, fewer patients in this group had received adjuvant chemotherapy compared with group B, which might also lead to worse outcome.

Minor issues

1. Authors may add regimen of different adjuvant chemotherapy in each group.
2. How can authors distinguish local recurrence from metachronous colon cancers (line 149)?
3. In line 280, authors mentioned an analysis comparing the four chemotherapy regimens showed no significant differences in DFS among them. Can you provide the reference?
4. In this study, authors should define current smoker and former smoker more accurately. You can see a paper which was published at J Clin Oncol. 2013 Jun 1;31(16):2016-23. Especially former smoker, some patients might just stop smoking before operation. Another issue is previous smoking might already lead to lung injury, which may also lead to elevation of CEA.
