# Supplementary material for: Prognostic value of postoperative serum carcinoembryonic antigen levels in colorectal cancer patients who smoke
Source: PLoS One. 2020 Jun 5;15(6):e0233687. doi: 10.1371/journal.pone.0233687 (PMC7274431; doi:10.1371/journal.pone.0233687)
Supplement: Attachment — Submitted filename: comments to plos one.doc [file pone.0233687.s003.doc]

**General Comments:**

This is a generally well-written manuscript.

However, there are still some issues regarding scientific novelty, scientific interpretation of the results that need to be well-addressed.

Major comments:

1. In group C, the author defined elevated pre-operative and post-operative CEA group. However, in table1, the range of pre-operative CEA level is 2.8-263.5 ng/ml. Which means some patient’s CEA level was normal before operation.

The author should carefully defined the group C.

2. One important finding in this study is post-operative CEA level is significantly higher in smoking group than non-smoking group (2.6 vs. 3.1 ng/ml). Although the p value is significant, the difference is too small in clinical practice. My other question is in non-smoking group, the mean post-operative CEA level is 2.6 ng/ml, range from 0.5-84.9 ng/ml. However, in smoking group, the mean post-operative CEA level is 3.1 ng/ml, range from 0.5-12.5 ng/ml. It seems the distribution of CEA level in non-smoking group is very wide. Do the author have the confidence to claim the post-operative CEA level is really higher in smoking group than non-smoking group?

Minor comments:

In abstract, the post-operative CEA level in smoking group is 2.5 ng/ml. However, in the table 2, the level is 2.6 ng/ml.
