# Supplementary material for: Prognostic value of postoperative serum carcinoembryonic antigen levels in colorectal cancer patients who smoke
Source: PLoS One. 2020 Jun 5;15(6):e0233687. doi: 10.1371/journal.pone.0233687 (PMC7274431; doi:10.1371/journal.pone.0233687)
Supplement: Attachment — Submitted filename: Response_to_Reviewers.docx [file pone.0233687.s004.docx]

**Reviewer #1**

***Comments 1:***

*Different stage will lead to different outcome. In colon cancer, molecular markers such as K-Ras,N-Ras and B-Raf are also an important factors. This study included stage I, II and III colon-rectal cancer. Authors should list the distribution of stages within different groups. If possible, analysis of molecular subtypes are also important.*

**Response 1:**

Yes. We completely agree with the reviewer’s pertinent comments. The distribution of stages within different groups are now provided in the table below and in the revised manuscript (**Results section, Table 1, page 9**).

|  | Group A  (n = 152) | Group B  (n = 69) | Group C  (n = 52) | *p* |
| --- | --- | --- | --- | --- |
| TNM stage |  |  |  |  |
| I | 47 (31%) | 5 (7%) | 7 (13%) | < 0.001 |
| II | 62 (41%) | 29 (42%) | 19 (37%) |  |
| III | 43 (28%) | 35 (51%) | 26 (50%) |  |

We agree that the molecular markers play an important role in colorectal cancer prognosis. However, under consideration of cost, molecular tests were only reserved for patients with stage IV disease in our hospital. The patients included in this study lacked molecular profiles such as *KRAS*, *NRAS,* and *BRAF*. We added this as a limitation in the revised manuscript (**Discussion section, page 19, lines 295-297**).

***Comments 2:***

*2. In line 223, authors mentioned about groups C had the worse outcome. But in group C, there were more patients with right side tumors compared with Group B and A, and more patients were LN positive compared with Group A. In summery, group C was already with worse prognostic factors, nothing to do with CEA level before and after operation. Interesting, fewer patients in this group had received adjuvant chemotherapy compared with group B, which might also lead to worse outcome.*

**Response 2:**

Yes. We agree with the reviewer’s insightful concern. Tumor sides, tumor stage, and chemotherapy are all well-known prognostic factors of colorectal cancer. As shown in Fig 1a, we found that in the CEA group, the overall 3-year DFS rate was significantly higher in group A (84.2%), with respect to group B (74.3%) and C (62.0%). Univariate analysis in our study showed that the CEA group was predictive of disease-free survival.

**Univariate analysis of the CEA group for disease-free surviva**l

|  | No. of patients | 3-year DFS rate | *p* |
| --- | --- | --- | --- |
| **CEA group** |  |  |  |
| **Group A** | 152 | 84.2% | 0.001 |
| **Group B** | 69 | 74.3% |  |
| Group C | 52 | 64.7% |  |

If we select the CEA group rather than the preoperative and postoperative CEA as a variable, CEA group, tumor stage, and age were significant independent prognostic factors for disease-free survival in multivariable analysis.

**Multivariate analysis of prognostic factors for disease-free survival**

|  | | Hazard ratio | 95% CI | | *p* | | |
| --- | --- | --- | --- | --- | --- | --- | --- |
| **CEA group** | |  |  | |  | | |
| **Group A** | | 1 |  | |  | | |
| **Group B** | | 1.299 | 0.760–2.222 | | 0.338 | | |
| **Group C** | | 2.356 | 1.369–4.060 | | 0.002 | | |
| **TNM stage** |  | | |  | |  |  |
| **I** | 1 | | |  | |  |  |
| **II** | 2.085 | | | 0.797–5.457 | | 0.135 |  |
| **III** | 4.603 | | | 1.791–11.828 | | 0.002 |  |
| **Age, years** |  | | |  | |  |  |
| **< 75** | 1 | | |  | |  |  |
| ≥ 75 | 1.643 | | | 1.057–2.553 | | 0.028 |  |

As a result, we believe that tumor side, stage, chemotherapy, and elevated postoperative CEA may all have contributed to the poorer prognosis in group C.

***Comments 3:***

*Authors may add regimen of different adjuvant chemotherapy in each group.*

**Response 3:**

We thank the reviewer for the insightful comment. The regimen of different adjuvant chemotherapy within different groups are provided in the tables below and in the revised manuscript

**Regimen of adjuvant chemotherapy within CEA groups**

|  | Group A  (n = 152) | Group B  (n = 69) | Group C  (n = 52) | *p* |
| --- | --- | --- | --- | --- |
| Regimen |  |  |  |  |
| FOLFOX | 54 (36%) | 36 (52%) | 16 (31%) | < 0.001 |
| XELOX | 0 (0%) | 2 (3%) | 0 (0%) |  |
| UFUR | 35 (23%) | 19 (28%) | 18 (34%) |  |
| Capecitabine | 0 (0%) | 0 (0%) | 2 (4%) |  |
| No | 63 (41%) | 12 (17%) | 16 (31%) |  |

**(Results section, Table 1, page 9)**

**Regimen of adjuvant chemotherapy within smoking groups**

|  | NS (n = 199) | S (n = 74) | *p* |
| --- | --- | --- | --- |
| Regimen |  |  |  |
| FOLFOX | 77 (39%) | 29 (39%) | 0.843 |
| XELOX | 2 (1%) | 0 (0%) |  |
| UFUR | 52 (26%) | 20 (27%) |  |
| Capecitabine | 2 (1%) | 0 (0%) |  |
| No | 66 (33%) | 25 (34%) |  |

**(Results section, Table 2, page 12)**

***Comments 4:***

*How can authors distinguish local recurrence from metachronous colon cancers?*

**Response 4:**

We thank the reviewer for the thoughtful comment. Local recurrence was observed in 13 patients in our study. Most of these cases initially had locally advanced cancer with adjacent organ involvement. The clinical features of patients with local recurrence are shown in the table below.

| Case No. | Recurrent Site | Synchronous Metastasis |
| --- | --- | --- |
| 1 | Pelvis | Nil |
| 2 | Pelvis | Liver |
| 3 | Abdominal wall | Paraaortic lymph nodes |
| 4 | Pelvis | Liver |
| 5 | Pelvis | Liver |
| 6 | Abdominal wall | Nil |
| 7 | Psoas muscle | Liver |
| 8 | Abdominal wall | Nil |
| 9 | Anastomosis | Ovary |
| 10 | Bladder | Nil |
| 11 | Anastomosis | Lung |
| 12 | Abdominal wall | Nil |
| 13 | Abdominal wall | Carcinomatosis |

Based on the clinical features we presented above, these cases could be distinguished from metachronous colon cancer.

***Comments 5:***

*In line 280, authors mentioned an analysis comparing the four chemotherapy regimens showed no significant differences in DFS among them. Can you provide the reference?*

**Response 5:**

Yes. We thank the reviewer for the insightful comments. Univariate analysis of chemotherapy regimens for disease-free survival are provided in the table below and in the revised manuscript

**Univariate analysis of chemotherapy regimens for disease-free survival**

|  | No. of patients | 3-year DFS rate | *p* |
| --- | --- | --- | --- |
| **Regimen** |  |  |  |
| **FOLFOX / XELOX** | 108 | 76.3% | 0.506 |
| UFUR / Capecitabine | 74 | 73.4% |  |

The sample sizes of those on XELOX and capecitabine were too small for conclusions. As a result, we compared between FOLFOX/XELOX versus UFUR/Capecitabine instead. (**Results section, page 13, lines 203-206**)

***Comments 6:***

*In this study, authors should define current smoker and former smoker more accurately. You can see a paper which was published at J Clin Oncol. 2013 Jun 1;31(16):2016-23. Especially former smoker, some patients might just stop smoking before operation. Another issue is previous smoking might already lead to lung injury, which may also lead to elevation of CEA.*

**Response 6:**

Yes. We agree with the reviewer’s astute concern, some patients might just have stopped smoking before the operation, and previous smoking might have already led to lung injury. However, the lung function and CEA levels could significantly improve after 3 months of smoke cessation [1]. Thus, we placed former smokers in the group NS along with never smokers, rather than in the S group, which included patients who smoked both before and after the surgery.

We agree that CEA levels are supposed to be different between never smokers and former smokers. However, one of the limitations of our study include the relatively small number of former smokers (n = 26), which likely resulted in limited statistical power. The amount and duration of smoking and type of cigarette would be taken into consideration in our future studies.

1. Pezzuto A, Spoto C, Vincenzi B, Tonini G. Short-term effectiveness of smoking-cessation treatment on respiratory function and CEA level. J Comp Eff Res. 2013;2: 335-343.

***Reviewer #2***

***Comments 1:***

*According to your statistic method, I don’t think it support your conclusion and the title. You should focus on the patients with persistent elevated CEA and using NS/S to compare disease-free survival if prognostic factors are balanced between two groups.*

**Response 1:**

Yes. We thank the reviewer for the thoughtful comments. We did compare disease-free survival between smoker and nonsmoker patients with persistent elevated CEA (**Results section page 12,** in line 189). In subgroup analysis, smokers had higher 3 year-DFS rates than nonsmokers in group C (83.3% vs. 43.9%, p = 0.029; Figure 2).

According to your suggestion, we have revised our title for the manuscript. Further, we also added more descriptions and two figures below and in the revised manuscript to demonstrate the 3 year-DFS rates between smokers and nonsmokers in different subgroups.

“The overall 3-year DFS rate was similar between smokers and nonsmokers (80.2% vs. 77.1%, *p* = 0.485; Fig 2a). In the subgroup analysis, the 3-year DFS rate was also similar between smokers and nonsmokers in groups A and B (78.8% vs. 81.8%, *p* = 0.853; Fig 2b). In contrast, smokers had higher 3 year-DFS rate than nonsmokers in group C (83.3% vs. 43.9%, *p* = 0.029; Fig 2c).” **(Results section, pages 12-13, lines 189-193)**

**Fig 2. Disease-free survival rates between smokers and nonsmokers** (a) Overall disease-free survival (DFS) rates. (b) patients with normal postoperative serum CEA levels. (c) patients with elevated postoperative serum CEA levels.

***Reviewer #3***

***Comments 1:***

*In group C, the author defined elevated pre-operative and post-operative CEA group. However, in table1, the range of pre-operative CEA level is 2.8-263.5 ng/ml. Which means some patient’s CEA level was normal before operation. The author should carefully defined the group C.*

**Response 1:**

Yes. We thank the reviewer for the thoughtful comments. There were 6 patients with normal preoperative CEA and elevated postoperative CEA in group C. One of them had tumor recurrence as carcinomatosis, 10 months after curative surgery. The clinical features of the patients are shown in the table below.

| Case No. | Preoperative CEA | Postoperative CEA | Recurrence |
| --- | --- | --- | --- |
| 1 | 4.8 | 7.4 | Carcinomatosis |
| 2 | 4 | 6.1 | Nil |
| 3 | 2.8 | 6.5 | Nil |
| 4 | 3.7 | 9.3 | Nil |
| 5 | 3.9 | 6.4 | Nil |
| 6 | 4.1 | 5.1 | Nil |

According to your suggestion, we have redefined group C as patients with elevated postoperative CEA in the revised manuscript.

***Comments 2:***

*One important finding in this study is post-operative CEA level is significantly higher in smoking group than non-smoking group (2.6 vs. 3.1 ng/ml). Although the p value is significant, the difference is too small in clinical practice. My other question is in non-smoking group, the mean post-operative CEA level is 2.6 ng/ml, range from 0.5-84.9 ng/ml. However, in smoking group, the mean post-operative CEA level is 3.1 ng/ml, range from 0.5-12.5 ng/ml. It seems the distribution of CEA level in non-smoking group is very wide. Do the author have the confidence to claim the post-operative CEA level is really higher in smoking group than non-smoking group?*

**Response 2:**

Yes. We thank the reviewer for the thoughtful comments. We have used Mann-Whitney U test to compare the postoperative CEA between smokers and nonsmokers. Although there are few mild outliers and extreme outliers among the nonsmoker group, the difference in the median postoperative CEA between smokers and nonsmokers was statistically significant (*p* = 0.009). The distribution of postoperative CEA is shown in the box and whisker plot below.

Although the absolute median difference between the two groups is relatively small, the proportion of patients with elevated postoperative CEA is significantly higher in the smoker group (32% vs. 14%, *p* <0.001). However, smokers with elevated postoperative CEA levels had a higher 3 year-DFS rates than non-smoking patients with similar postoperative CEA levels (83.3% vs. 43.9%, p = 0.029). These findings support our conclusion that postoperative serum CEA levels showed no prognostic efficacy for smoker patients

***Comments 3:***

*In abstract, the post-operative CEA level in smoking group is 2.5 ng/ml. However, in the table 2, the level is 2.6 ng/ml.*

**Response 3:**

Yes, we thank the reviewer for the kind reminder. We have corrected the abstract data in the revised manuscript.
